# Case report: A complex case of valve-in-valve TAVI and left bundle branch pacing for severe aortic regurgitation with partially corrected type A aortic dissection and low ejection fraction

**DOI:** 10.3389/fcvm.2023.1206811

**Published:** 2023-08-10

**Authors:** Peter Marko Mihailovič, David Žižek, Luka Vitez, Primoz Holc, Tomislav Klokočovnik, Matjaž Bunc

**Affiliations:** ^1^ Department of Cardiology, University Medical Center Ljubljana, Slovenia; ^2^ Department of Cardiovascular Surgery, University Medical Center Ljubljana, Slovenia; ^3^ Medical Faculty, University of Ljubljana, Slovenia

**Keywords:** transcatheter aortic valve implantation (TAVI), valve in valve transcatheter aortic valve implantation, valve in valve implantation, aortic regurgitation (AR), heart failure, cardiac resychronisation therapy, left bundle area pacing, left bundle branch pacing (LBBP)

## Abstract

**Background:**

Aortic regurgitation is a major concern following transcatheter aortic valve implantation (TAVI), as even low-grade regurgitation is associated with increased mortality. This is of particular concern to patients with pre-existing aortic disease who are at increased risk of TAVI valve slippage. Furthermore, conduction system disturbances after TAVI, namely left bundle branch block (LBBB), may have an additional detrimental effect on cardiac function.

**Case presentation:**

This report documents a successful treatment strategy in a frail patient with a bicuspid aortic valve and aortic disease after valve-sparing surgical repair in 1998, who subsequently developed aortic stenosis and underwent TAVI with an Evolut R self-expanding aortic valve. The progression of aortic disease, aortic root dilatation, and leaflet degeneration over the following years caused aortic regurgitation of the self-expanding aortic valve, resulting in left ventricular dilatation and heart failure along with LBBB and left ventricular (LV) mechanical dyssynchrony. Diagnostic workup of the patient showed persistence of the aneurysm distal to the graft with a dissection spanning the ascending aorta, arch, and terminating proximal to the aortic isthmus. After consideration by the cardiac team, a balloon-expandable valve was chosen for a valve-in-valve (ViV) procedure to provide sufficient radial force to expand the existing valve and correct the regurgitation. Due to the anatomy, a J-wire and pigtail catheter were successfully used for a safe approach and placement of the valve. Following the procedure, intermittent complete atrioventricular block was observed in addition to the pre-existing left bundle branch block, necessitating resynchronization pacing. Due to anatomical considerations, ease of placement, and the expected good level of resynchronization due to the proximal block, we opted for left bundle branch pacing, which showed improvement in left ventricular dyssynchrony and LV function at follow-up.

**Conclusion:**

Valve-in-valve implantation of a balloon-expandable Myval TAVI device to treat aortic regurgitation caused by slippage and right leaflet disfunction of slef valve is feasible in challenging anatomical scenarios. Left bundle branch pacing is a viable alternative to correct mechanical dyssynchrony in complex patients with LBBB and anatomical challenges necessitating resynchronization.

## Introduction

Aortic regurgitation following transcatheter aortic valve implantation (TAVI) is an important concern, as even low-grade paravalvular leak (PVL) is associated with increased mortality (1). This is especially important in patients with pre-existing aortic disease, who are at inherent risk for PVL and TAVI valve slippage due to unfavorable characteristics such as larger-diameter annuli, complex valve shapes, and progressive aortopathy (2). TAVI can also lead to conduction system disturbances, namely left bundle branch block (LBBB), which has detrimental effects on cardiac function due to impaired ventricular mechanics and cardiac remodeling (3). We outline a complex case of an elderly frail patient with partially corrected aortic disease and persistent aortic dissection, dilatative heart failure, and aortic regurgitation on a previously implanted self-expanding transcatheter heart valve, along with mechanical dyssynchrony due to left bundle branch block.

## Case description

### Presentation

**Figure 1:**
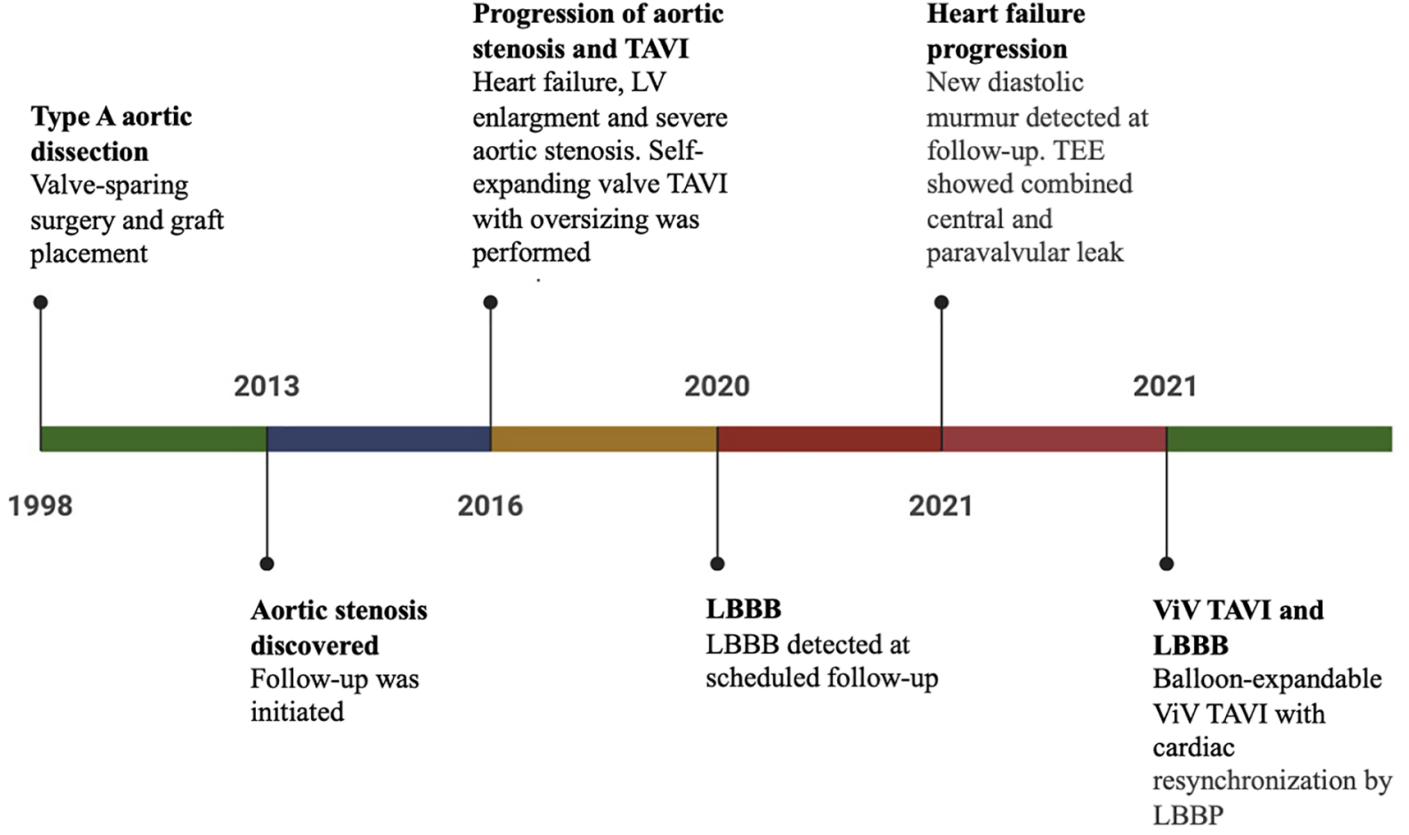
Timeline of care.

A 79-year-old female patient was referred to our center due to severe aortic insufficiency after a previously implanted transcatheter aortic valve (TAVI). The patient's complex medical history included a bicuspid aortic valve with an aneurysm of the ascending aorta, which resulted in an acute type A aortic dissection in 1998. Following the dissection, emergency aortic valve reconstruction and a valve-sparing aortic root procedure (David procedure) were performed. In 2013, aortic stenosis and heart failure (HF) were diagnosed, both of which deteriorated during subsequent visits to the outpatient clinic. A Bentall procedure was a suboptimal therapeutic option due to the high procedural risk and the possibility of complications. TAVI was chosen as the intervention of choice in 2016, with planned oversizing due to the expected dilatation of the aortic root. An Evolut R 29 mm self-expanding valve was successfully inserted via a transfemoral approach with good expansion but low placement and minimal paravalvular leak seen after the procedure. Left bundle branch block (LBBB) developed after the first TAVI procedure. The patient was admitted again to a regional hospital shortly before referral to our center in 2021 due to worsening HF symptoms. Echocardiography confirmed a severely reduced ejection fraction (EF 20%) with concomitant severe aortic valve insufficiency resulting from a severe paravalvular leak. The patient was transferred to our center for further evaluation of possible treatment options (patient characteristics available in Supplementary S1, Timeline in Figure 1).

### Examination

BMI 20.44 kg/m^2^, BSA 1.38 m^2^, afebrile, pulse rate of 75 bpm, respiratory rate of 21/min, and blood pressure of 112/65 mmHg. Blood oxygen saturation was normal without supplemental oxygen therapy. Lower extremity pretibial edema was present.

## Diagnostic assessment

### Laboratory tests

NT-pro BNP of 17,098 ng/L, creatinine 59 umol/L.

**The ECG** showed sinus rhythm with a left bundle branch block with a QRS width of 230 ms (Supplementary Figure S2).

**Figure 2:**
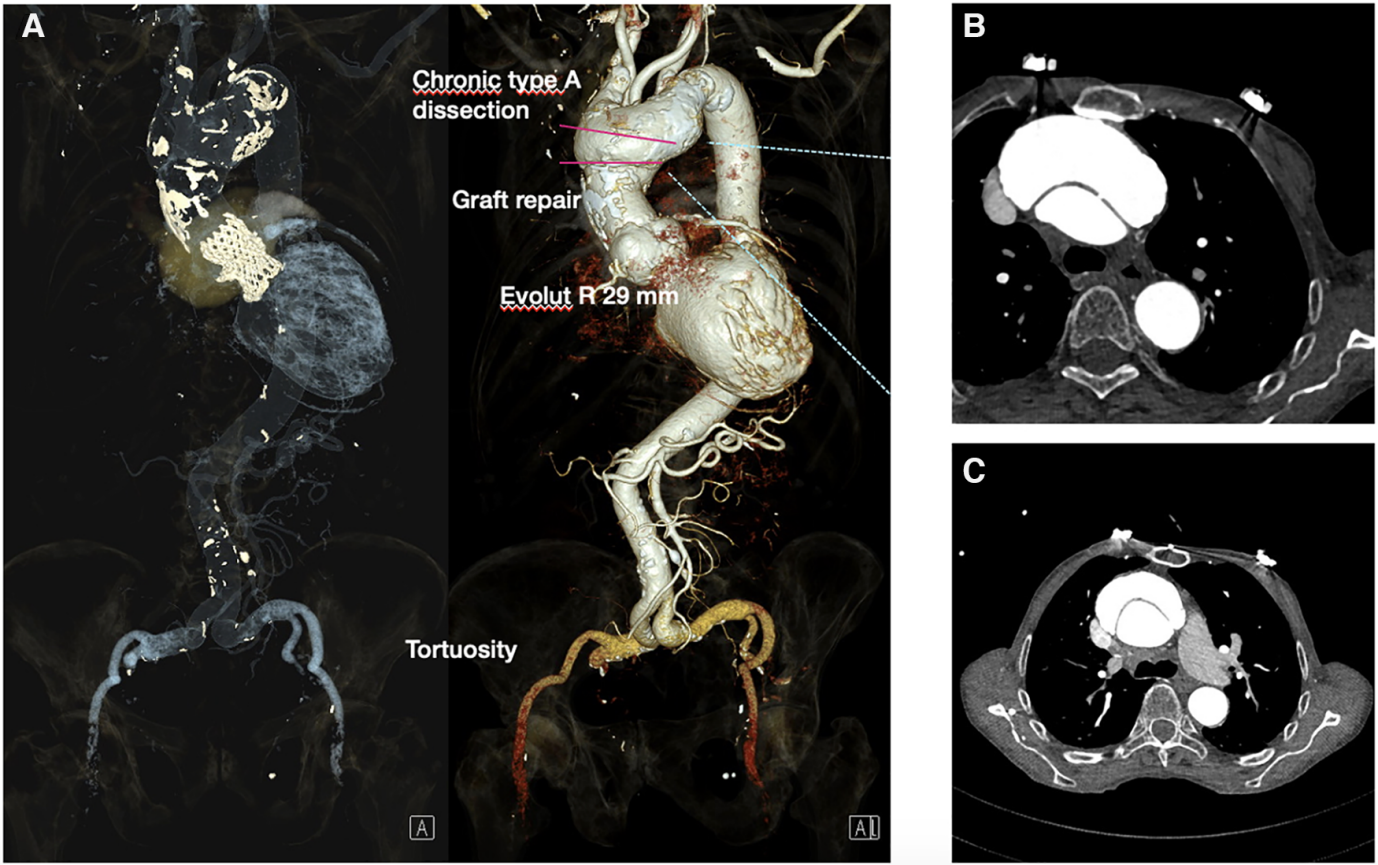
CT angiography following TAVI protocol: (**A**) EvolutR TAVI valve with graft repair and aortic aneurysm with type A dissection of the ascending aorta and proximal arch. Severe tortousity of the iliac arteries. (**B,C**) Type A aortic dissection in the transverse plane.

**Echocardiography** revealed an enlarged left ventricle (EDD 6.4 cm, EDV 305 ml, EDVI 221 ml/m^2^) with mild wall hypertrophy (IVS 1.1 cm, inf-lat 1.4 cm) with severely reduced ejection fraction, a normal stroke volume (LVEF 22%, SV 70 ml), and severe valvular and paravalvular insufficiency of the Evolut R biological TAVI valve with mild mitral regurgitation. The central valvular insufficiency was caused by the degeneration of the right leaflet, which led to a diagnostic work-up and exclusion of infective endocarditis of the artificial valve—this was ruled out with transesophageal ultrasound (TEE), cardiac magnetic resonance imaging (MRI), and positron emission tomography (PET-CT) scans, in addition to serial blood cultures, which remained sterile. Echocardiographic signs of LV mechanical dyssynchrony were present. These findings were also confirmed by MRI. A CT of the thoracic and abdominal aorta revealed persistence of the ascending aortic aneurysm distal to the graft placement with dissection spanning the ascending aorta and aortic arch and terminating proximal to the aortic isthmus with involvement of the brachiocephalic trunk and progression of the aortic root dilatation compared to previous CT scans (Figure 2, Supplementary Figures S3, S4). Severe tortuosity of the iliac arteries was evident upon 3D reconstruction. Coronary angiography showed no significant atherosclerotic lesions. Infective endocarditis was excluded as the cause of the valve degeneration.

### Procedure

**Figure 3:**
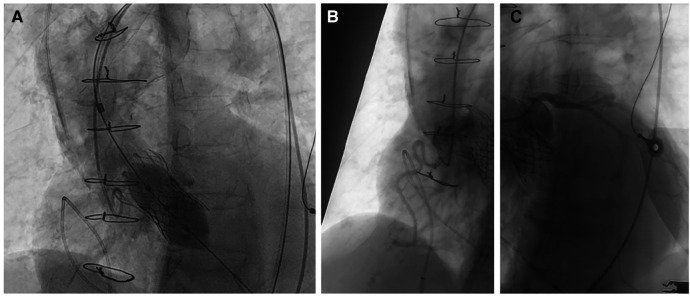
(**A**) Myval 26 mm positioning no predilation. Good angiographic result with no regurgitation. The position of the valve was at the level of the natural annulus. We avoided overextension of the neo-skirt. (**B,C**) Unobstructed coronary ostia with good patency.

Due to the frailty of the patient and the high surgical risk, the cardiac team's consensus was to correct the valve regurgitation with a valve-in-valve TAVI using a Myval 26 mm (Meril, Gujarat, India) valve due to the appropriate size, the radial force achieved by balloon-expandable valves, and the shorter stent frame height of the valve, reducing the neo-skirt. Careful procedural planning was performed in order to assess the anatomical considerations of the slipped Evolut R valve and its relationship to the aortic root. A bifemoral approach was obtained by positioning a 16F transcatheter aortic valve introducer in the right femoral artery and a 6F introducer for a pigtail catheter in the left femoral artery. Due to the severe iliac artery tortuosity, a nitinol hydrophilic guide wire covered with polyurethane Radifocus ™guidewire M (Terumo, Tokyo, Japan) with RJ 4.0 6F support was used. A distal part “S-reshaped” J soft wire-mounted pigtail catheter was used to safely cross the dissected segment (Supplementary Figure S5). The aortic valve was then crossed with a straight tip soft wire using an Amplatz left (AL) 1 catheter (Launcher, Medtronic, Minnesota, USA). The soft wire was exchanged for an extra-stiff 0.0035” Lunderquist wire (Cook Medical, Indiana, USA). We then proceeded with the implantation of a 26 mm Myval valve, which was placed without predilation of the existing Evolut R (Supplementary Figure S6). The Myval valve was positioned at the level of the natural bicuspid aortic valve ring (10/90, outflow/aortic bulbus). Control selective coronary angiography showed good patency of the coronary arteries, and control aortography showed good alignment of the prosthetic valve with no residual aortic regurgitation (Figure 3).

### Pacemaker implantation

**Figure 4:**
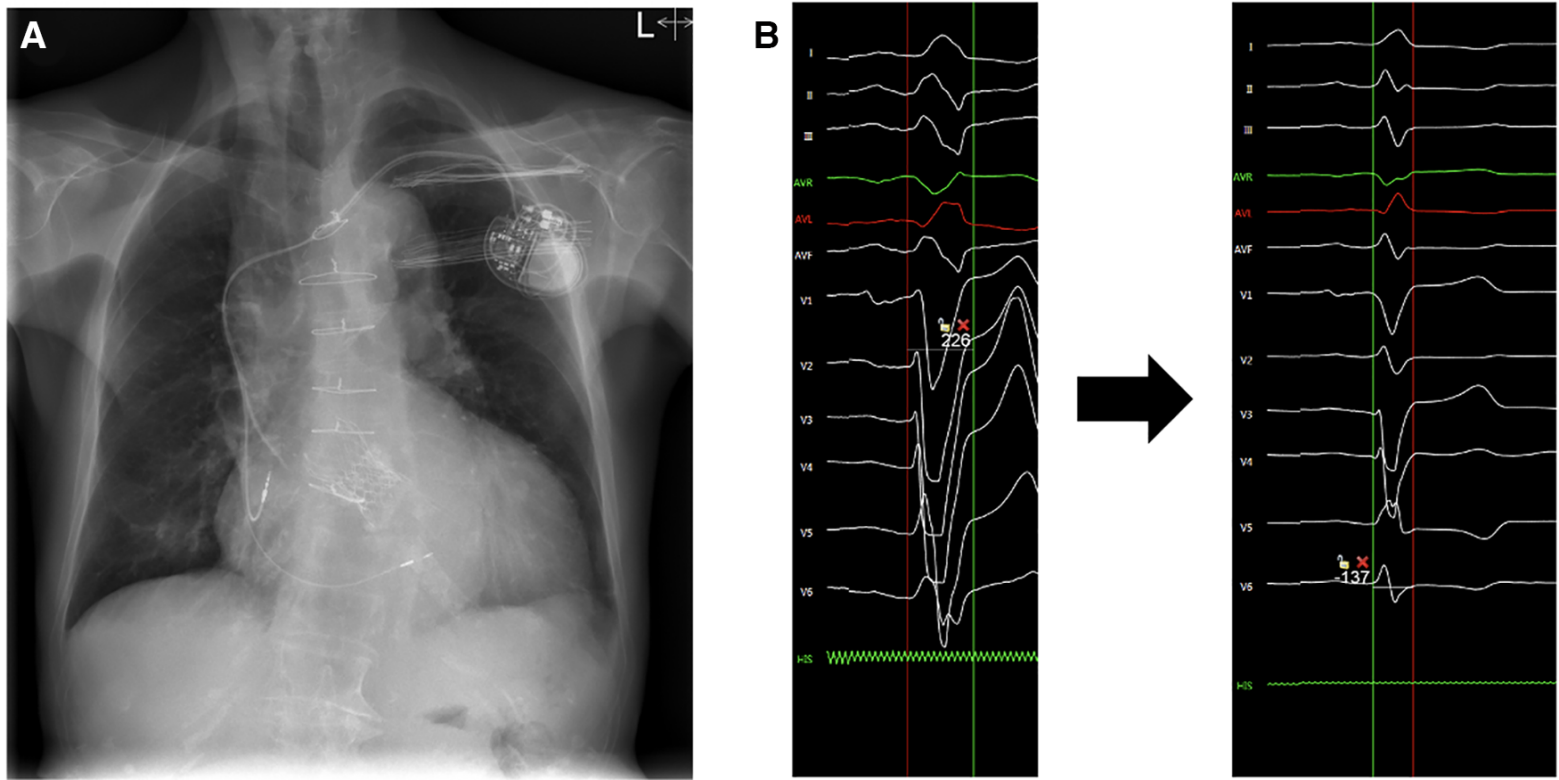
(**A**) Postoperative x-ray of the dual-chamber pacemaker with the atrial lead positioned in the right atrial appendage and the ventricular lead positioned transeptally for left bundle branch pacing (LBBP). (**B**) (Left) Initial QRS complex with left bundle branch block morphology. (Right) Final QRS duration after atrio- ventricular delay optimization.

Following valve-in-valve TAVI, intermittent complete AV block was recorded. As LBBB was already present after the first TAVI procedure in conjunction with severely reduced ejection fraction, resynchronization therapy was indicated. As an alternative to biventricular pacing, we opted for the LBBAP technique. A right atrial lead (Capsurefix MRI Surescan 52 cm, Medtronic) was temporarily inserted into the right ventricle (RV) to prevent potential asystole due to mechanical injury of the right bundle branch during LBBP lead positioning. As previously described (14–18), a 3,830 SelectSecure 69 cm lead (Medtronic) and a C315His (Medtronic) catheter were used for transseptal lead insertion. Selective left bundle branch capture with correction of the left bundle branch block was achieved (Supplementary Figures S5, S6). The right atrial lead was then positioned in the RA, and the atrioventricular delay was optimized to achieve the shortest QRS duration (Figure 4).

### Post-procedure echocardiography

A post-procedural echocardiography did not show immediate improvement in left ventricular function with a residual dilatation of the left ventricle (EDV 333 ml, EDVI 247 ml/m^2^), severely reduced ejection fraction (EF 17%) with a normal stroke volume (SV 74 ml), elevated LV filling pressures, and moderate post-capillary pulmonary hypertension. The TAVI valve was well expanded with minimal trace paravalvular leakage. A Doppler ultrasound of the bifemoral puncture sites was performed before discharge due to a murmur, which did not reveal signs of fistulas or pseudoaneurysms.

### Follow-up

Follow-up in the cardiology clinic revealed functional improvement in the patient with some residual exertional dyspnea but minimal signs of heart failure (NYHA class II). Follow-up echocardiography was also performed and showed improvement in LV function with an increase in ejection fraction (3D EF 27% as compared to 17% before discharge, Supplementary S7) with some minimal residual signs of mechanical dyssynchrony and normal filling pressures of the left ventricle, minimal paravalvular and central aortic regurgitation, and mild tricuspid regurgitation adjacent to the ventricular pacemaker lead with normal pulmonary pressures. Laboratory markers of HF also improved, with an NT-proBNP of 4,089 ng/L.

## Discussion

Structural valve degeneration and paravalvular leaks are not uncommon after TAVI. According to the literature, redo TAVI for the treatment of acute post-procedural or late paravalvular regurgitation is associated with favorable clinical and echocardiographic outcomes (4). In our patient, the deterioration was most likely due to a combination of progression of the native aortic disease with dilatation of the aortic root that resulted in TAVI valve slippage and degeneration of one of the valve leaflets, resulting in central valve regurgitation. After ruling out infective endocarditis and after consideration by the cardiac team, a ViV TAVI procedure using a balloon-expandable device was selected due to the higher radial expansion force and the hope of also expanding the underlying stent frame of the Evolut R valve and correcting the paravalvular leak. The Myval balloon-expandable valve was chosen for its design—the small cell lower part provides higher radial strength, and the small size and low skirt reduce the chances of coronary artery occlusion and minimize neo-skirt formation. Along with this, the external PET skirt provides good sealing for any paravalvular leak (5). The case was challenging for several reasons. First, the tortuosity of the iliac arteries proved to be a challenge for peripheral access. For this reason, a Lunderquist® Extra Stiff wire was used for support; due to its linear stiffness characteristics (6), the Lunderquist® wire is often the preferred wire for large endograft delivery (7) and is used when greater support is required to deliver the TAVI device due to aortic tortuosity (8). Second, the presence of an aortic dissection spanning the entire aortic arch required a non-traumatic approach that would lower the risk of rupture. For this, a mounted pigtail catheter was used to safely cross the dissected segment using a technique used for thoracic endovascular aortic repair (TEVAR) of dissection (9). The case also highlights the strengths and advantages of using a balloon-expandable valve-in-valve TAVI. This is one of the few published attempts at a Myval valve-in-valve TAVI procedure for aortic valve insufficiency and proves that the use of a Myval valve for this kind of procedure is feasible. The valve was positioned according to valve implantation recommendations for bicuspid valve implantation. We targeted the natural annulus of the bicuspid valve in addition to the closed-cell portion of the Evolut R valve. Another important aspect of this case is the choice of pacing modality. According to data, 3.8%–20% of patients develop atrioventricular heart block, necessitating pacemaker implantation after TAVI (10). Standard RV pacing is associated with worsening HF and increased mortality (11). TAVI patients with permanent pacemakers experience more postoperative HF admissions. There was also a trend toward increased mortality; this was especially notable in patients with >40% RV pacing (12). Furthermore, standard RV pacing after TAVI was associated with negative effects on LVEF (13). Left bundle branch pacing (LBBP) promises to be a more physiological pacing modality compared to standard RV pacing for bradycardia and resynchronization indications (14, 15). In a recent study of LBB pacing following prosthetic valve implantation, the procedure proved to be feasible (16). In addition, LBBP pacing is associated with higher implant success rates and more stable pacing parameters compared to the other physiologic pacing modality, His bundle pacing (17). Although not yet validated by large randomized controlled trials, LBB pacing is proving to be a simpler alternative to other pacing modalities, including biventricular pacing (18). Our patient developed intermittent complete AV block following the valve-in-valve TAVI procedure in addition to the previously known LBBB. According to guidelines, patients with HF and LBBB require a cardiac resynchronization therapy (CRT) device with an implantable cardioverter-defibrillator (ICD). The decision to opt for LBBP instead of standard biventricular pacing was based on several factors. First, the patient was very frail and had a very low BMI, which precluded the use of devices with bulkier batteries. Second, LBBB was likely to be induced by TAVI; therefore, LBBP could be performed for resynchronization as the proximal block was expected (19), using only one lead and the smallest possible dual-chamber pacemaker. Third, correction of LBBB with transeptal lead positioning to reach the conduction system resulted in complete QRS normalization and acute improvement of mechanical dyssynchrony, with further improvement of EF during follow-up.

## Patient perspectives and conclusion

This complex case report highlights the feasibility of using a Myval transcatheter prosthetic valve for redo TAVI to correct paravalvular regurgitation and valve degeneration in a patient with pre-existing aortic disease. In addition, LBBP can be used as an alternative resynchronization pacing modality following TAVI in frail patients with a low BMI and concerns about device size.

## Data Availability

The original contributions presented in the study are included in the article/**Supplementary Material**, further inquiries can be directed to the corresponding author.

## Supplementary material

Supplementary Video S1 Left ventricular function before ViV TAVI and LBBP.

Supplementary Video S2 Left ventricular function after ViV TAVI and LBBP.

Supplementary Video S3 Aortic regurgitation prior to ViV TAVI.
