# Supplementary figures and images for: Case report: A complex case of valve-in-valve TAVI and left bundle branch pacing for severe aortic regurgitation with partially corrected type A aortic dissection and low ejection fraction
Source: Front Cardiovasc Med. 2023 Aug 10;10:1206811. doi: 10.3389/fcvm.2023.1206811 (PMC10449538; doi:10.3389/fcvm.2023.1206811)

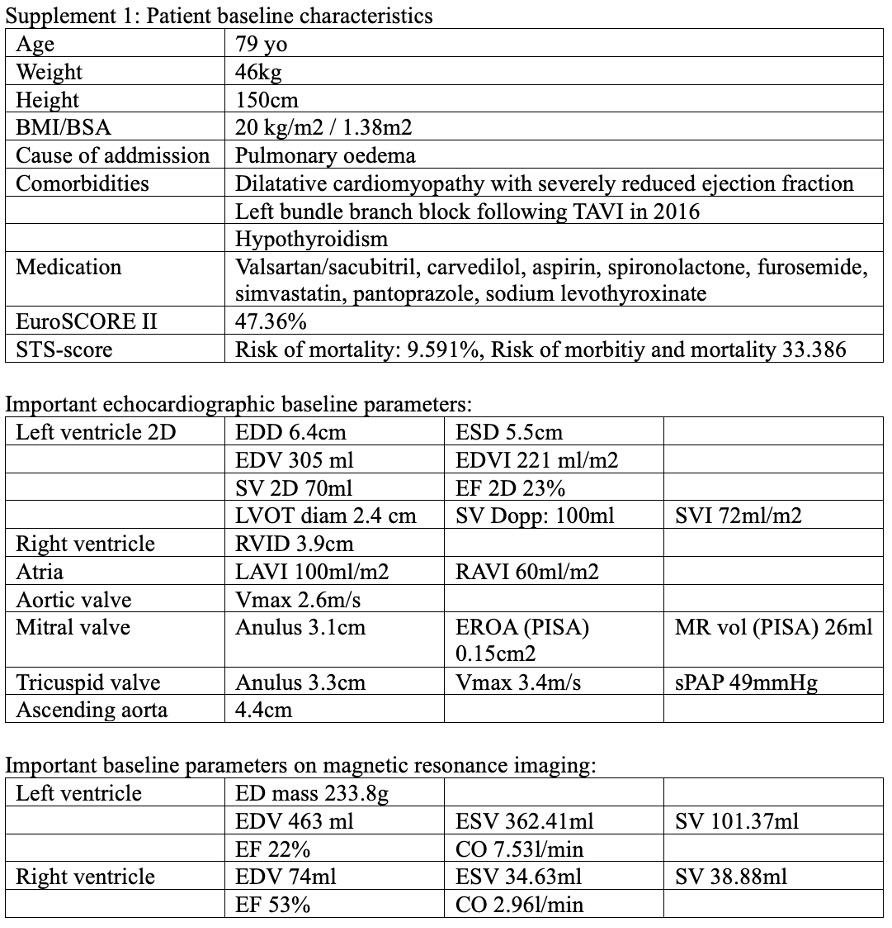

Supplement: Supplementary file 4 [file Image1.jpeg]

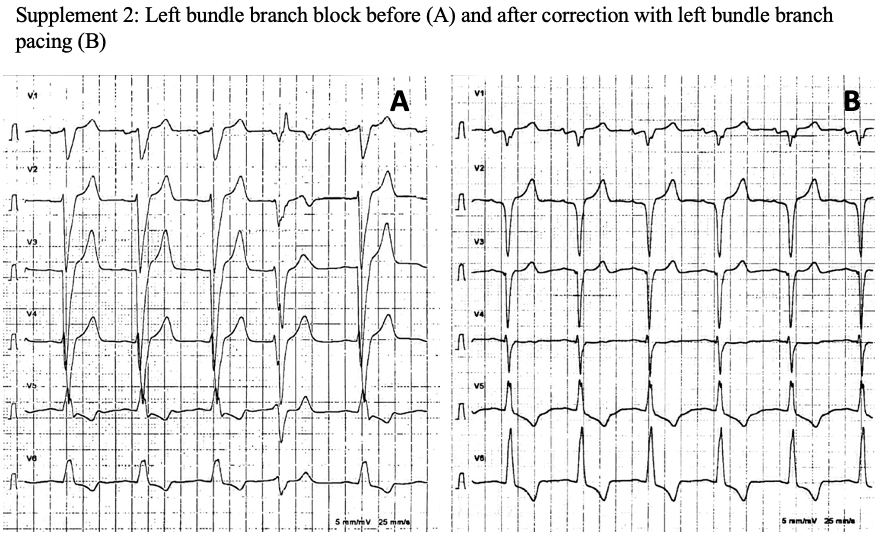

Supplement: Supplementary file 5 [file Image2.png]

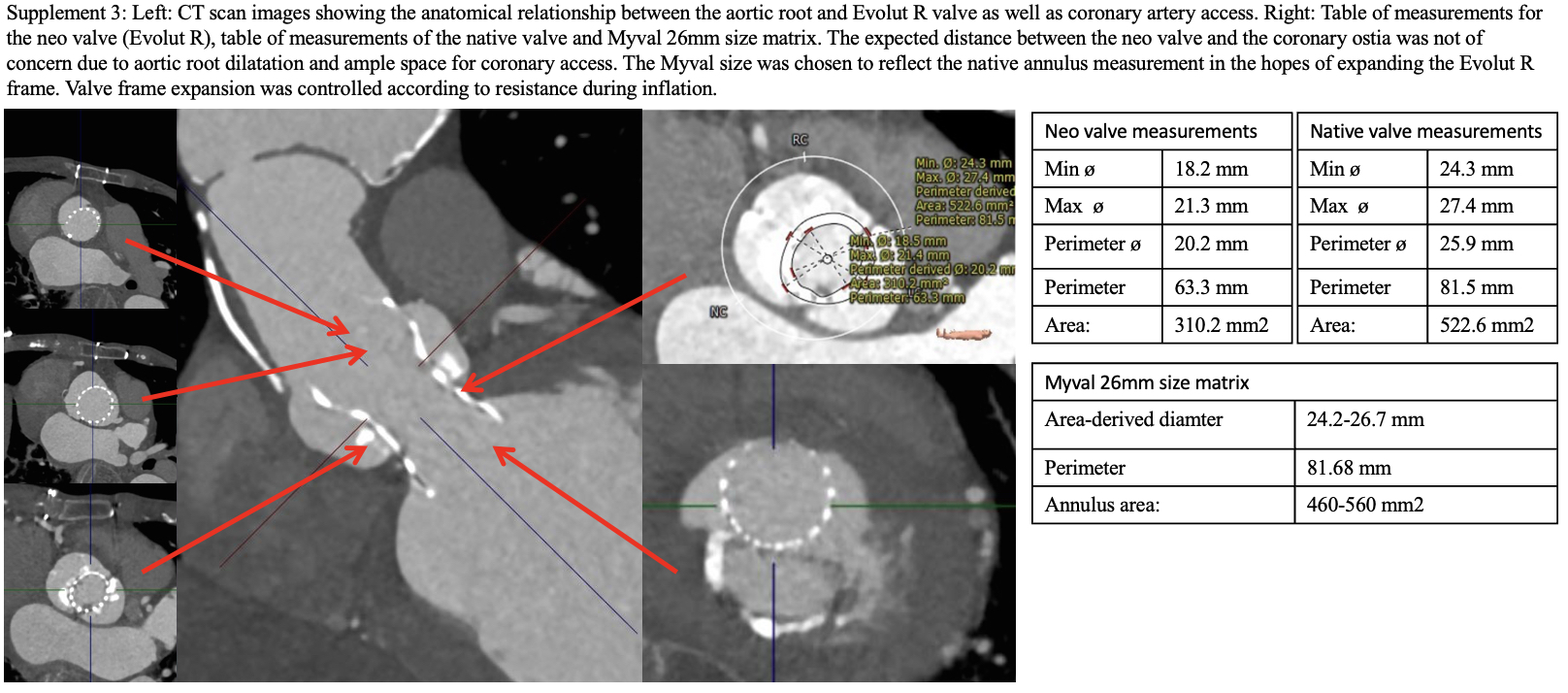

Supplement: Supplementary file 6 [file Image3.jpeg]

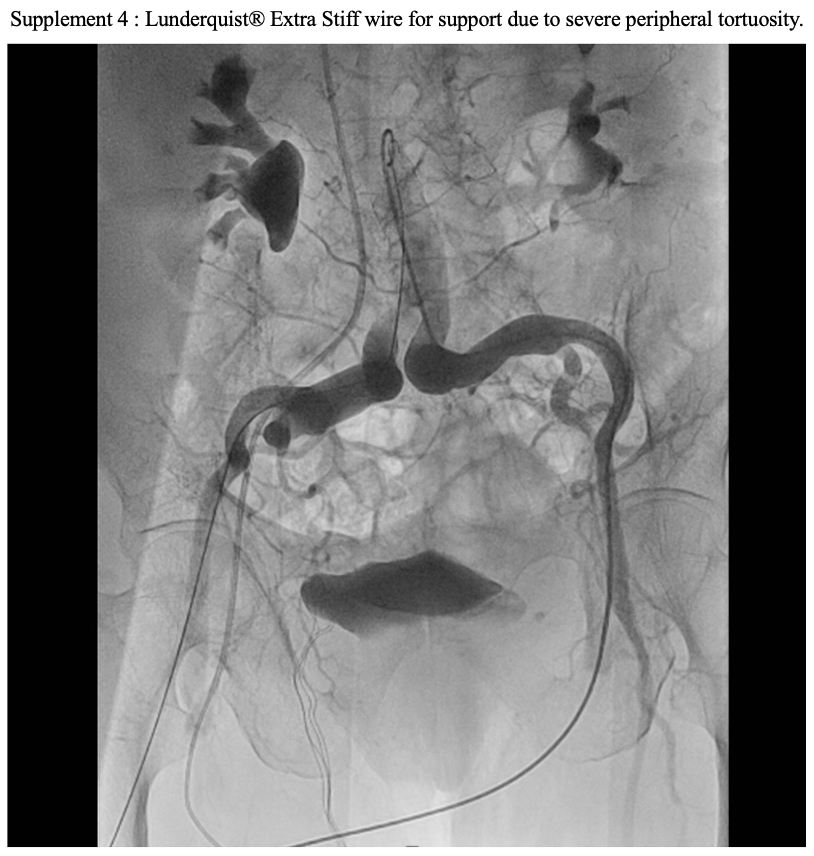

Supplement: Supplementary file 7 [file Image4.jpeg]

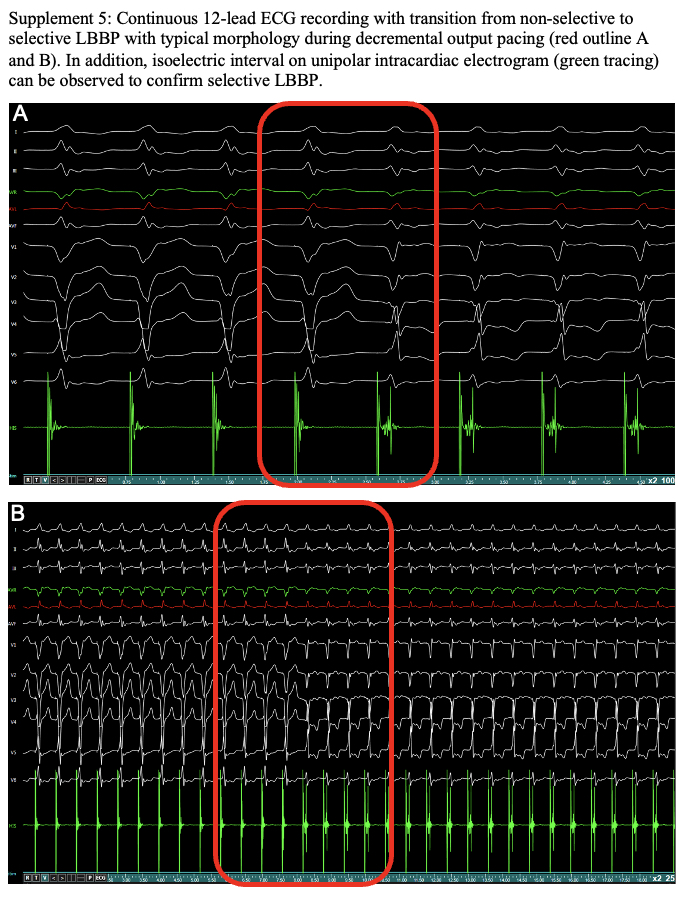

Supplement: Supplementary file 8 [file Image5.jpeg]

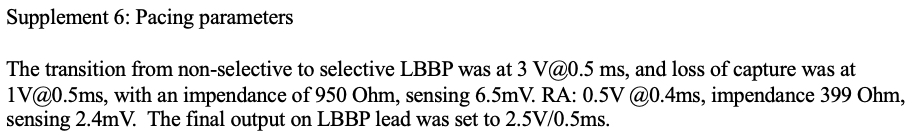

Supplement: Supplementary file 9 [file Image6.jpeg]

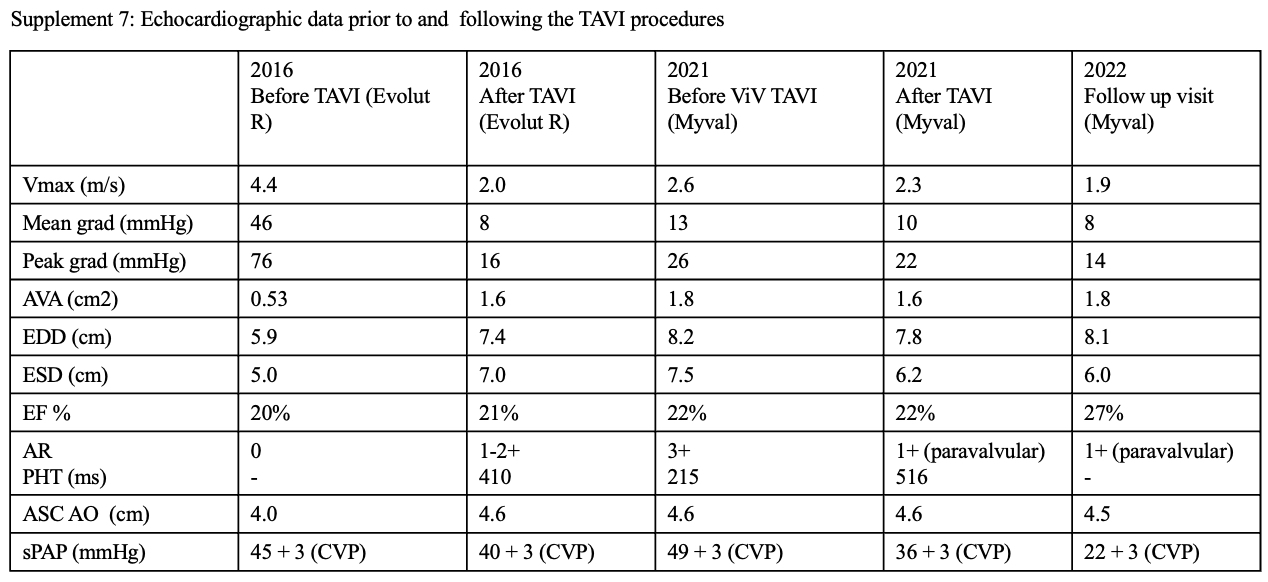

Supplement: Supplementary file 10 [file Image7.jpeg]
